# Supplementary material for: The use of transcranial ultrasound and clinical assessment to diagnose ischaemic stroke due to large vessel occlusion in remote and rural areas
Source: PLoS One. 2020 Oct 2;15(10):e0239653. doi: 10.1371/journal.pone.0239653 (PMC7531787; doi:10.1371/journal.pone.0239653)
Supplement: S1 Table — (DOCX) [file pone.0239653.s001.docx]

**S1 Table. Sensitivity analyses of the regression models for likely diagnosis of intracranial haemorrhage and acute ischaemic stroke due to LVO based on transcranial ultrasound and clinical assessment.**

| 1. ***SENSITIVITY ANALYSES FOR DIAGNOSIS OF INTRACRANIAL HAEMORRHAGE*** | | | | |
| --- | --- | --- | --- | --- |
| 1. ***Diagnostic accuracy of the prognostic model within 24 hours from symptom onset to transcranial ultrasound*** | | | | |
| **Observed diagnosis** | | **Predicted diagnosis** | | |
|  |  | Ischaemic stroke, TIA or stroke mimics | ICH | Percentage correct |
| Ischaemic stroke, TIA or stroke mimics | | 40 | 0 | 100 |
| ICH | | 3 | 4 | 57 |
| **Overall percentage correct = 94%** | | | | |
| Sensitivity = 57%; specificity = 100%; PPV = 100%; NPV = 93% | | | | |
| 1. ***Diagnostic accuracy of the prediction model after exclusion of cases with insufficient acoustic window*** | | | | |
| **Observed diagnosis** | | **Predicted diagnosis** | | |
|  |  | Ischaemic stroke, TIA or stroke mimics | ICH | Percentage correct |
| Ischaemic stroke, TIA or stroke mimics | | 71 | 1 | 99 |
| ICH | | 6 | 8 | 57 |
| **Overall percentage correct = 92%** | | | | |
| Sensitivity = 57%; specificity = 99%; PPV = 89%; NPV = 92% | | | | |
| 1. ***Diagnostic accuracy of the prediction model after inclusion of cases with insufficient acoustic window into the regression model using theoretical results of present or absent signs of intracranial haemorrhage on transcranial ultrasound based on the final diagnosis*** | | | | |
| **Observed diagnosis** | | **Predicted diagnosis** | | |
|  |  | Ischaemic stroke, TIA or stroke mimics | ICH | Percentage correct |
| Ischaemic stroke, TIA or stroke mimics | | 80 | 1 | 99 |
| ICH | | 6 | 12 | 67 |
| **Overall percentage correct = 93%** | | | | |
| Sensitivity = 67%; specificity = 99%; PPV = 92%; NPV = 93% | | | | |
| 1. ***Diagnostic accuracy of prediction model after exclusion of cases treated with IV tPA prior to transcranial ultrasound scan from the regression model*** | | | | |
| **Observed diagnosis** | | **Predicted diagnosis** | | |
|  |  | Ischaemic stroke, TIA or stroke mimics | ICH | Percentage correct |
| Ischaemic stroke, TIA or stroke mimics | | 60 | 1 | 98 |
| ICH | | 6 | 10 | 63 |
| **Overall percentage correct = 91%** | | | | |
| Sensitivity = 63%; specificity = 98%; PPV = 91%; NPV = 91% | | | | |
| 1. ***SENSITIVITY ANALYSES FOR DIAGNOSIS OF ACUTE ISCHAEMIC STROKE DUE TO LVO*** | | | | |
| 1. ***Diagnostic accuracy of the prediction model within 24 hours of symptom onset to transcranial ultrasound*** | | | | |
| **Observed diagnosis** | | **Predicted diagnosis** | | |
|  |  | Acute ischaemic stroke due to LVO | Acute ischaemic with no signs of LVO; stroke mimics and TIAs | Percentage correct |
| Acute ischaemic stroke due to LVO | | 6 | 4 | 60 |
| Acute ischaemic stroke with no signs of LVO; stroke mimics and TIAs | | 0 | 32 | 100 |
| **Overall percentage correct = 91%** | | | | |
| Sensitivity = 60%; specificity = 100%; PPV = 100%; NPV = 89% | | | | |
| 1. ***Diagnostic accuracy of the prediction model after exclusion of cases with insufficient acoustic window from the model*** | | | | |
| **Observed diagnosis** | | **Predicted diagnosis** | | |
|  |  | Acute ischaemic stroke due to LVO | Acute ischaemic with no signs of LVO; stroke mimics and TIAs | Percentage correct |
|  | Acute ischaemic stroke due to LVO | 6 | 5 | 55 |
|  | Acute ischaemic stroke with no signs of LVO; stroke mimics and TIAs | 2 | 62 | 97 |
| **Overall percentage correct = 91%** | | | | |
| Sensitivity = 55%; specificity = 97%; PPV = 75%; NPV = 93% | | | | |
| 1. ***Diagnostic accuracy of prediction model after inclusion of cases with insufficient acoustic window into the regression model using theoretical results of present or absent signs of LVO on transcranial ultrasound*** | | | | |
| **Observed diagnosis** | | **Predicted diagnosis** | | |
|  |  | Acute ischaemic stroke due to LVO | Acute ischaemic with no signs of LVO; stroke mimics and TIAs | Percentage correct |
|  | Acute ischaemic stroke due to LVO | 7 | 6 | 54 |
|  | Acute ischaemic stroke with no signs of LVO; stroke mimics and TIAs | 3 | 73 | 96 |
| **Overall percentage correct = 90%** | | | | |
| Sensitivity = 54%; specificity = 96%; PPV = 70%; NPV = 92% | | | | |
| 1. ***Diagnostic accuracy of prediction model after exclusion of cases treated with IV tPA prior to transcranial ultrasound scan from the regression model*** | | | | |
| **Observed diagnosis** | | **Predicted diagnosis** | | |
|  |  | Acute ischaemic stroke due to LVO | Acute ischaemic with no signs of LVO; stroke mimics and TIAs | Percentage correct |
|  | Acute ischaemic stroke due to LVO | 4 | 3 | 57 |
|  | Acute ischaemic stroke with no signs of LVO; stroke mimics and TIAs | 2 | 55 | 97 |
| **Overall percentage correct = 92%** | | | | |
| Sensitivity = 57%; specificity = 96%; PPV = 67%; NPV = 95% | | | | |
| *Predicted probability cut-off value is 50%. Abbreviation: ICH – intracranial haemorrhage; IV tPA – intravenous thrombolysis with tissue plasminogen activator; LVO – large vessel occlusion; NPV – negative predictive value; PPV – positive predictive value; TIA – transient ischaemic attack.* | | | | |
